# Associations between graduated driver licensing and road trauma reductions in a later licensing age jurisdiction: Queensland, Australia

**DOI:** 10.1371/journal.pone.0204107

**Published:** 2018-09-25

**Authors:** Teresa Senserrick, Soufiane Boufous, Jake Olivier, Julie Hatfield

**Affiliations:** 1 Transport and Road Safety Research, School of Aviation, The University of New South Wales, Sydney, New South Wales, Australia; 2 School of Mathematics and Statistics, The University of New South Wales, Sydney, New South Wales, Australia; UCL, UNITED KINGDOM

## Abstract

The success of driver graduated licensing systems (GLS) is demonstrated primarily in jurisdictions that licence at young ages with requirements expiring at age 18. In Australia, GLS requirements typically apply for all applicants aged under 25. In 2007, the Queensland licensing system was strengthened, extending the learner and introducing a 100-hour supervised driving requirement, introducing restrictions on passenger carriage at night and high-powered vehicles for provisional drivers, and on phone use for all novice drivers (learner and provisional). The objective of the current research was to evaluate whether these changes were associated with reductions in crashes (all) and killed-and-serious-injury (KSI) crashes involving novice drivers, and respective casualties. Government licensing and police crash records were linked and interrupted time series analysis was used to examine potential shifts in crash trends by rates of licensed drivers per month. Substantial declines were found in novice driver crashes (13.1% per year; 95%CI -0.0130, -0.0096), crash casualties (13.9% per year; 95%CI -0.0137, -0.0101), KSI crashes (5.4% per year; 95%CI -0.0080, -0.0046) and associated casualties (5.2% per year; 95%CI -0.0075, -0.0039). Compared to the total licensed driver population, declines in crashes (3.0% per year; 95%CI -0.0027, -0.0007) and crash casualties (2.9% per year; 95%CI -0.0029, -0.0006) but not KSI outcomes were observed. More narrowly, declines were found for provisional-licensed driver crashes (9.3% per year; 95%CI -0.0096, -0.0063) and KSI crashes (3.6% per year; 95%CI -0.0004, -0.0128) that were approximately 2.6% and 1.2% greater than respective declines for 25-29-year-old open-licensed drivers. Substantial declines also were observed in novice driver single-vehicle, night, passenger and alcohol crashes. Overall, these results demonstrate that GLS can be effective in a later age licensing jurisdiction. However, KSI outcomes were limited. Modelling research is recommended on ways to further strengthen Queensland’s GLS to achieve greater trauma reductions.

## Introduction

In the state of Queensland (QLD), Australia, young newly-licensed drivers are over-represented in road trauma statistics, as found globally [1]. For example, in 2015, provisional-licensed drivers and riders aged 16–24 years comprised just 5.2% of the licensed population in QLD [2] but were involved in crashes resulting in 21.8% of all fatalities and 32.0% of all hospitalised casualties [3]. The QLD government has long aimed to attenuate young novice crash risk by implementing a graduated licensing system (GLS) in which new drivers must first meet requirements of a supervised learner period, then a restricted provisional (unsupervised) licence period from a minimum age of 17 years, before progressing to an open (unrestricted) licence from a minimum age of 19 years, with at least some requirements applying to all applicants aged under 25.

International evaluations have identified this licensing approach as extremely effective in reducing crashes and injuries [4–5]. However, the majority of GLS evaluations have been conducted in the United States where generally independent driving ages of 16 years or younger can apply and requirements are only applicable if aged under 18. To date, there has not been a complete evaluation of an Australian GLS in which later licensing ages apply. This is primarily as Australian jurisdictions have relatively smaller populations, so that several years of post-introduction data is required to detect differences in crash and injury rates [6]. Additionally, most previous GLS evaluations have focused on potential reductions in young driver crashes or casualty crashes, rather than reductions in the number of actual casualties (i.e. people injured, which could include the driver, passenger and/or other road users) in these crashes, which are arguably the more important metric in determining the value of such interventions.

**Fig 1:**
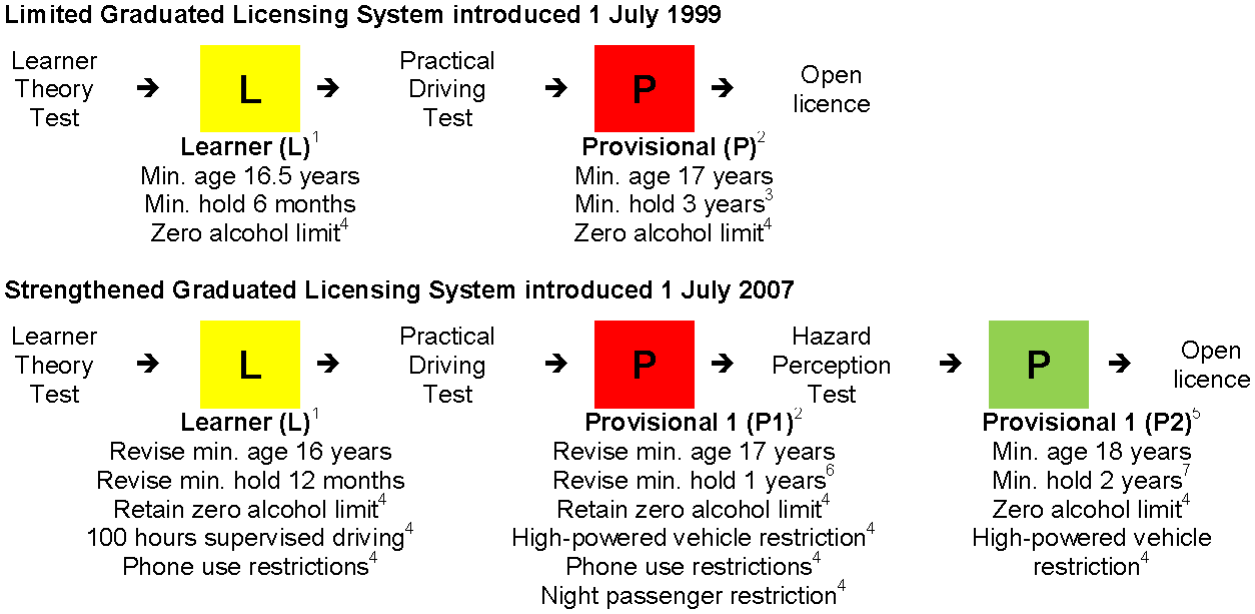
Summary of Queensland’s graduated licensing system. ^1^ Drivers must be supervised and display a yellow L-plate; ^2^ Drivers must display a red P-plate; ^3^ If aged <25 years; ^4^ If aged <23 years, else two years if aged 23 or one year if aged ≥24; ^5^ Drivers must display a green P-plate; ^6^ If aged <25 years, else advance directly to P2; ^7^ If P1 issued when aged <23 years and now aged <25 years, else one year if aged ≥25 or if P1 issued at age 23 and now aged ≥24 years, otherwise if P1 issued at age 24+ advance directly to open licence.

While QLD had elements of a traditional three-stage GLS in place since 1 July 1999, its GLS requirements and restrictions were strengthened from 1 July 2007 with the specific aim of reducing the number of people killed or seriously injured in crashes involving a novice driver (with serious injury determined by admission to hospital). The changes included a shift from a three- to a four-stage system involving two phases of provisional licence: the first year as “P1” and following two years as “P2”. This allowed additional restrictions in the first year, identified as the period of highest risk for newly-licensed drivers, not only in QLD but elsewhere in Australia and internationally [7]. A summary of QLD’s GLS before and after the 2007 changes is presented in Fig 1.

The main changes to the learner stage were a six-month extension of the minimum period (to 12 months) with a corresponding six-month reduction in the minimum learner age (to 16 years). A requirement for 100 hours of logged supervised practice driving was introduced along with restriction from use of any phone functions (including hands-free) by drivers and from use of loudspeaker functions by passengers. These phone restrictions were also introduced for the P1 stage along with a night passenger restriction: only one passenger aged under 21 years from 11pm to 5am (excluding family). A high-powered vehicle restriction was introduced across the three provisional years (i.e. P1 and P2). These changes applied only to novice drivers aged under 25 years. However, from 1 July 2010, the zero alcohol limit that already applied to under-25 year-old novice drivers prior to the 2007 changes was extended to apply to all new learner and provisional drivers (regardless of age).

Other aspects of QLD’s GLS are of note but were not specifically evaluated. The introduction of the 100 hours logbook requirement for learners included a requirement for a minimum of 10 hours at night and also a “3-for-1” scheme. Under this scheme, one hour with a professional driving instructor can count as three hours for the first 10 hours of lessons; therefore there is the potential to reduce the minimum logbook hours to 80 hours. Additional conditions that applied to novice drivers both before and after the GLS changes included a reduced demerit point threshold for traffic offences (4 points compared to 12 points for open-licensed drivers). For all drivers irrespective of licence type, if the threshold is reached the driver has a choice of a three-month licence suspension or a one-year “good driving behaviour” period, during which no more than one demerit point can be accumulated. If breached, a six-month suspension applies. Further, for provisional drivers (only) under a good driving behaviour condition or returning to driving following a licence suspension or disqualification (including as a result of reaching the demerit point threshold), a late night driving restriction applied from 11pm to 5am.

A preliminary evaluation of the potential impact on QLD’s revised GLS was conducted in 2009 (Newstead & Scully, 2013). The before-after study compared the crashes of novice (learner and provisional) drivers to open-licensed 25–35 year-olds over time, controlling for exposure by person months of licensure. Results suggested that the GLS achieved a 4% reduction in crashes, 13% reduction in fatal and serious injury crashes, and 30% reduction in fatal crashes, involving novice drivers. The authors concluded that more years of data were required for a full evaluation, particularly to determine the potential impact of the individual GLS changes. This was in part because during the transition from the old to new GLS special legislative allowances were made for anyone turning 16.5 years of age or applying for their first licence in the first six months following its introduction (to complete only a six-month minimum learner period and 60 hours of supervised driving). This resulted in multiple different GLS pathways at the time of the evaluation.

The objective of the current research was to evaluate whether the new GLS in QLD was achieving its goal of reducing the number of people killed or seriously injured through involvement in a novice driver crash. Associated changes in novice crashes (all) and in novice fatal and serious injury crashes were first examined in order to compare the results to previous international evaluations. These analyses were then repeated to examine changes in the casualty outcomes of these crashes. Further, changes in novice driver single-vehicle, night, passenger and alcohol-involved crashes were analysed.

## Methods

### Study design

The overall study design was to use interrupted time series analysis to assess any changes in crash outcomes involving a novice (learner or provisional licensed) driver comparing the years from commencement of GLS on 1 July 1999 through to the introduction of the new GLS on 1 July 2007 to available data post the new GLS up to 31 December 2015 (dependent on outcome as detailed below under ‘Data sources’). Trends were examined to explore changes in rates of licensed drivers per month, to account for different numbers of licence holders over time, in several ways:

1. Changes in crashes and killed or seriously injured (KSI) crashes involving novice drivers.
2. Changes in crashes and KSI crashes involving novice drivers relative to those of the remainder of the licensed driver population in Queensland.
3. Comparison of changes in crashes and KSI crashes involving provisional-licensed novice drivers only to those involving 25–29 year-old open-licensed drivers only.

The second set of analyses was included to act as a control for other potential influences on outcomes over time, such as general improvements to roads and vehicle occupant protection. The third set of analyses included a more narrow age control comparison. This was included to address a concern that the global economic downturn (which occurred around the same time the new GLS was introduced) might have particularly affected younger drivers [8].

Additionally, certain sub-types of novice crashes and KSI crashes were examined, including single-vehicle crashes (i.e. those involving only the novice driver vehicle) as a proxy for at-fault status. To explore the potential contributions of specific aspects of the new GLS, night crashes (11pm to 5am), passenger crashes (any passengers) and alcohol (BAC>0.00) crashes were also explored. There were too few crashes to analyse night passenger crashes specifically, and incomplete passenger age data did not allow analyses focused on passengers within a specific age-range. The trend analyses for novice driver crashes, KSI crashes and these crash sub-types were repeated in relation to the resulting casualties in the crash.

The study protocol was approved by the Human Research Ethics Advisory Panel H: Science and Engineering, The University of New South Wales (approval number HC16011). This study used de-identified, collated, state record data only, not requiring project-specific informed consent from individuals.

#### Data sources

Data were sourced from routinely collected licensing and police records maintained by the state government road authority, the Department of Transport and Main Roads. The TRAILS licensing database included records of when licences were acquired. Key variables for the current study included birth date, and start and end date (when applicable) of holding each licence (learner, provisional and open) for car drivers. (Records for other licence types, such as motorcycle and heavy vehicle, and any records showing that drivers originally obtained a licence outside of QLD were excluded.)

The Queensland Road Crash Database dataset included detailed information on police-recorded crashes. Key variables for the current study included the crash date and crash severity, with varying years of data available dependent on severity type. Data on fatal crashes, from which at least one person died within 30 days, were available to 31 December 2015, whereas data on serious injury crashes, those from which at least one person was admitted to hospital but no-one died, were available to 31 December 2013. Therefore, KSI crash analyses were limited to the end of 2013. Medical treatment crashes, from which at least one person sustained injury requiring medical treatment but no one was killed or seriously injured, and minor Injury crashes, those from which at least one person sustained a minor injury but no one was killed, seriously injured or had a medically treated injury, were available to 31 June 2012. Non-injury (property damage only) crashes, from which no one was injured, were available to 31 December 2010 only. Therefore, as including non-injury crashes would limit the years of data available in the new GLS period substantially, analyses of all crashes excluded the non-injury category (a category known to be under-reported to police regardless [9]).

As elements of the previous GLS were introduced from 1 July 1999, available records commencing from this date were the focus. Records from the TRAILS and Queensland Road Crash Database were linked by Transport and Main Roads for each level of crash severity using independent identifiers (based on licence numbers, de-identified to protect privacy). Each crash severity dataset was subsequently linked into a single dataset by the research team based on the Transport and Main Roads unique identifiers.

### Data analyses

To examine the overall trends in novice crashes over time relative to the start of the new GLS system on 1 July 2007, an interrupted time series analysis was conducted with the log of the ratios of numbers of licensed novice drivers (per 10,000) as an offset and also with all crashes as an offset. An autoregressive error process to account for serial correlation was estimated and this model had the form:

$$\text{log}\;y_{ti}\;=\;\beta_{0}+\beta_{1}t+\beta_{2}gls_{i}+\beta_{3}t\times gls_{i}+e_{ti}$$

where *e*_*ti*_ is the autoregressive process. The coefficient *β*_2_ is a measure of whether the rate “shifted” up or down and *β*_3_ is an indication if the trend changed with the introduction of the new GLS.

Separate models were fit for all novice driver crashes, proportion of novice driver crashes relative to all licensed drivers in QLD, provisionally-licensed driver crashes and 29 year-old open-licensed driver crashes and their respective KSI crashes, as well as for novice driver single-vehicle, night, passenger and alcohol crashes and KSI crashes. Models also were fit for corresponding analyses of crash casualties. Parameters were estimated by maximum likelihood and statistical inference was performed using likelihood-based methods. Tabulated summary statistics for analyses of crash trends for the licensed driver population include the estimate, 95% confidence intervals (CI) and p-values. The estimates “Change in Trend Post New GLS” corresponded to the estimates of monthly and yearly changes in rates, such that these were computed from the model estimate by:

%monthlychange=exp(Estimate)–1

%yearlychange=exp(12*Estimate)–1

All analyses were performed using SAS version 9.4 (SAS Institute, 2016) and R version “Sock it to me”.

## Results

### Trends in crashes and KSI crashes

**Fig 2:**
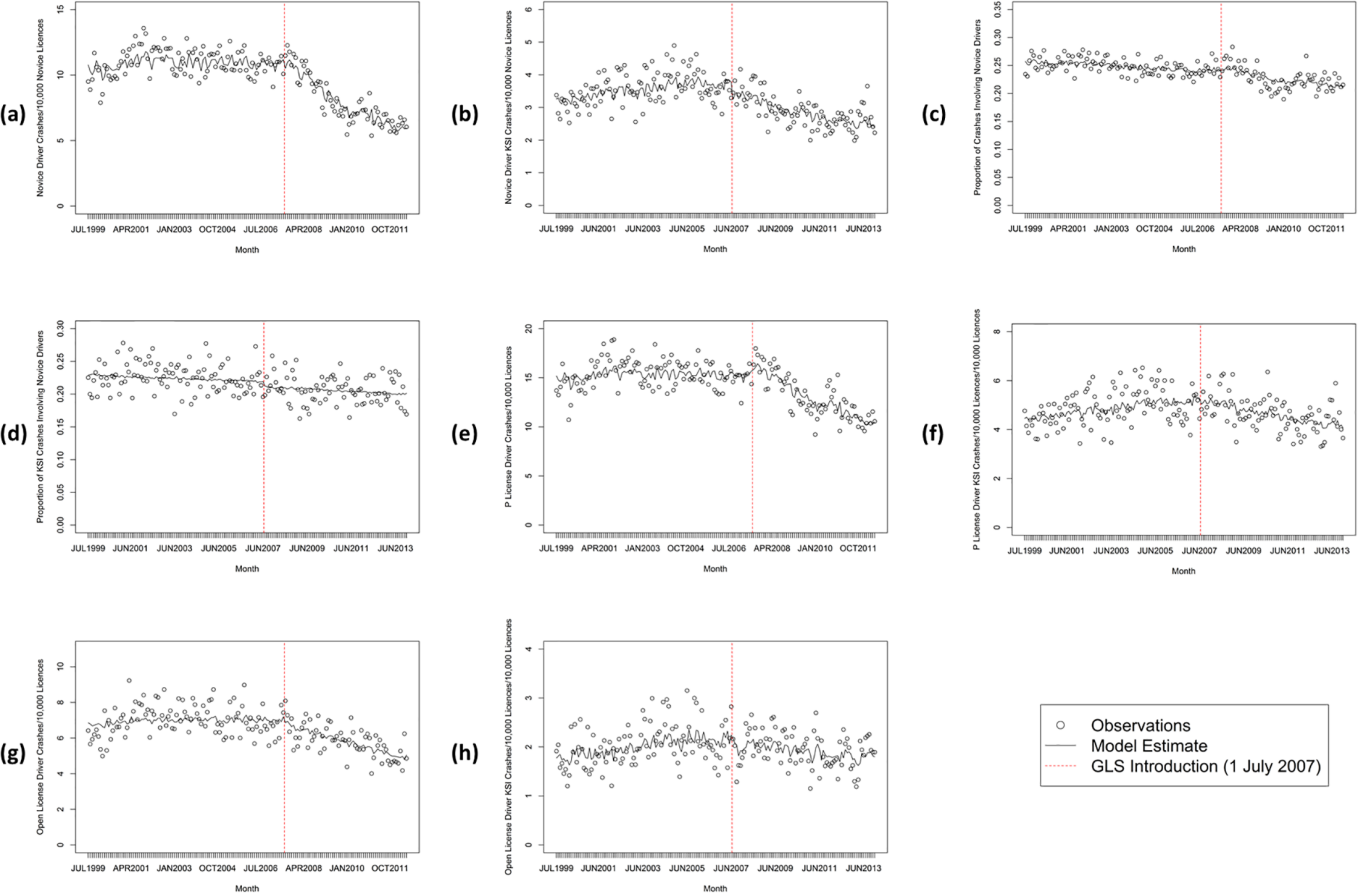
Crashes (1 July 1999–30 June 2012) and KSI crashes (1 July 1999–31 December 2013) involving novice drivers in Queensland, Australia. (a) crashes per 10,000 novice licences; (b) KSI crashes per 10,000 novice licences; (c) crashes as a proportion of all crashes; (d) KSI crashes as a proportion of all KSI crashes; (e) provisional-licensed driver crashes per 10,000 provisional licences; (f) provisional-licensed driver KSI crashes per 10,000 provisional licences; (g) 25–29 year-old open-licensed driver crashes per 10,000 respective licences; (h) 25–29 year-old open-licensed driver KSI crashes per 10,000 respective licences.

The overall trends in novice driver crashes and KSI crashes over time are depicted in Fig 2 while Table 1 presents summary statistics including the trends pre- and post-introduction of the new GLS and their confidence intervals and p-values. For all crashes involving novices per 10,000 novice licences [Fig 2(a)], the trend post the new GLS changed compared to the pre period. The post trend showed a decline in crashes at a rate of 1.1% per month or 13.1% per year. A change was also found for novice KSI crashes [Fig 2(b)], with a decline of 0.4% per month or 5.4% per year.

**Table 1:** Summary statistics of crashes^a^ and KSI crashes^b^ involving novice drivers in Queensland, Australia.

Time Series	Trend Pre New GLS				New GLS shift				Change in Trend Post New GLS			
	Estimate	95% CI		P	Estimate	95% CI		P	Estimate	95% CI		P
(a) Novice driver crashes^c^	0.0003	-0.0005	0.0010	0.4700	-0.0213	-0.0897	0.0471	0.5429	-0.0113	-0.0130	-0.0096	<.0001
(b) Novice driver KSI crashes^c^	0.0018	0.0008	0.0028	0.0004	-0.1361	-0.2180	-0.0542	0.0014	-0.0063	-0.0080	-0.0046	<.0001
(c) Proportion novice driver crashes	-0.0008	-0.0012	-0.0003	0.0011	0.0160	-0.0248	0.0568	0.4410	-0.0017	-0.0027	-0.0007	0.0016
(d) Proportion novice driver KSI crashes	-0.0005	-0.0012	0.0003	0.2035	-0.0364	-0.0960	0.0232	0.2334	-0.0003	-0.0015	0.0010	0.6850
(e) Provisional-licensed driver crashes^d^	0.0002	-0.0006	0.0009	0.6747	0.0483	-0.0178	0.1144	0.1533	-0.0080	-0.0096	-0.0063	<.0001
(f) Provisional-licensed driver KSI crashes^d^	0.0018	-0.0048	0.0065	0.0005	-0.0238	-0.2272	0.0106	0.5818	-0.0049	-0.0004	-0.0128	<.0001
(g) 25–29 year-old open licensed driver crashes^e^	0.0004	-0.0005	0.0012	0.4418	-0.0385	-0.1181	0.0411	0.3437	-0.0060	-0.0080	-0.0040	<.0001
(h) 25–29 year-old open-licensed driver KSI crashes^e^	0.0025	0.0011	0.0039	0.0006	-0.0886	-0.2048	0.0276	0.1371	-0.0045	-0.0069	-0.0022	0.0002

^a^ 1 July 1999 to 30 June 2012;

^b^ 1 July 1999 to 31 December 2013;

^c^ per 10,000 novice licences;

^d^ per 10,000 provisional licences;

^e^ per 10,000 25–29 year-olds’ open licences

For crashes involving novice drivers as a proportion of all crashes in QLD [Fig 2(c)], the trend post the new GLS changed compared to the pre period. The post-GLS rate of decline was 0.2% per month or 3.0% per year. However, the trend in the proportion of novice driver KSI crashes [Fig 2(d)] did not change in the post new GLS period compared to the pre period.

Focusing just on provisional-licensed drivers, the trend in crashes per 10,000 provisional licences [Fig 2(e)] following the new GLS changed compared to the pre period. A decline at a rate of 0.8% per month or 9.3% per year was found. A change was also found for provisional-licensed driver KSI crashes [Fig 2(f)], with a decline of 0.3% per month or 3.6% per year post the new GLS.

For the comparison group, the trend in crashes involving only drivers aged 25–29 years on an open licence per 10,000 respective licences also changed following the new GLS [Fig 2(g)]. A decline in crashes at a rate of 0.6% per month or 6.7% per year was found; therefore slower than the decline found for provisionally-licensed driver crashes by 0.2% per month or 2.6% per year. Likewise, a slower decline was found in the KSI crash trend of 25-29-year-old open-licensed drivers [Fig 2(h)]. A decline at a rate of 0.2% per month or 2.4% per year was found, indicating provisional-licensed driver KSI crashes declined by an additional 0.1% per month or 1.2% per year.

#### Trends in novice driver crash types

**Fig 3:**
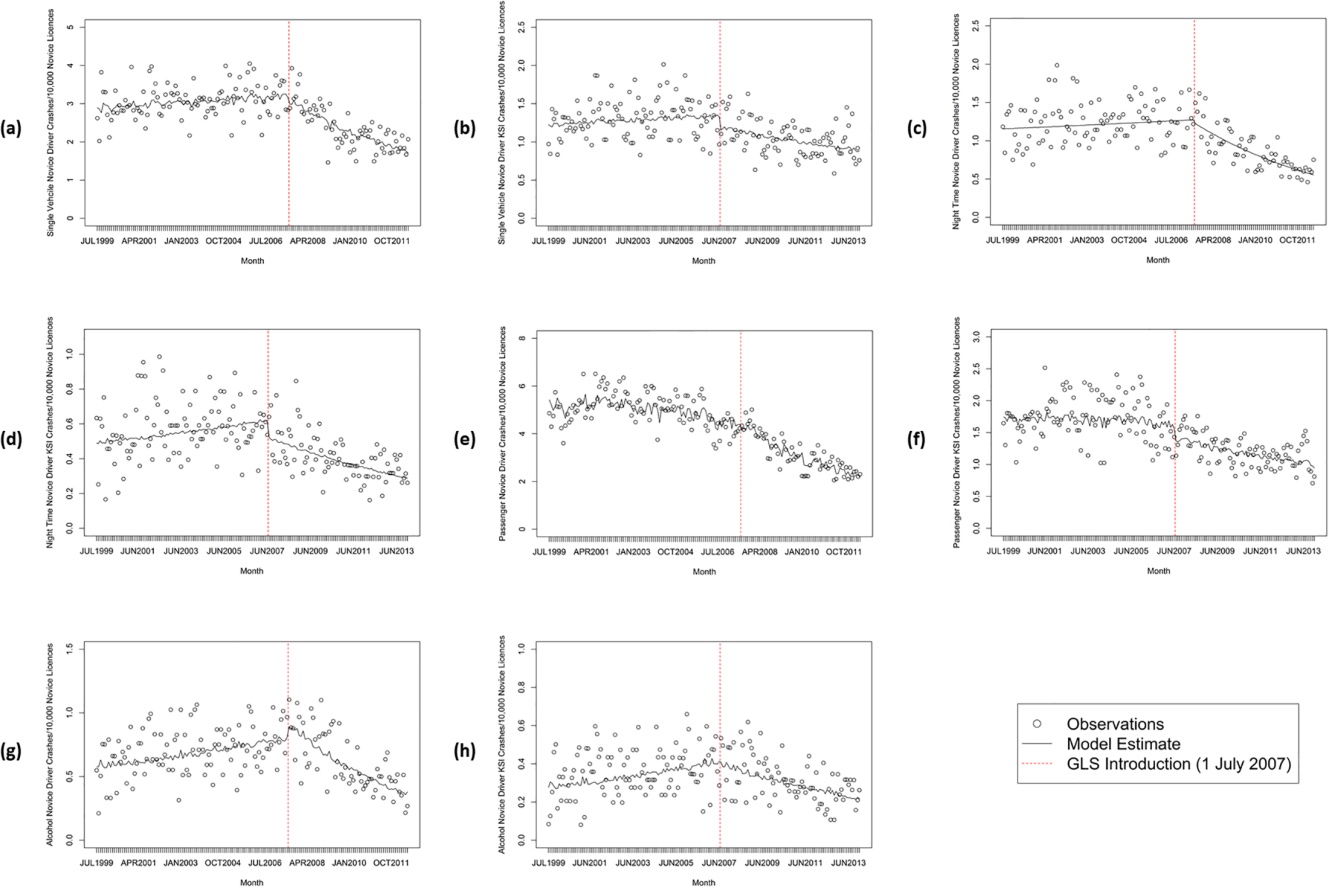
Types of crashes (1 July 1999–30 June 2012) and KSI crashes (1 July 1999–31 December 2013) involving novice drivers per 10,000 novice licences in Queensland, Australia. (a) single-vehicle crashes; (b) single-vehicle KSI crashes; (c) night (11pm to 5am) crashes; (d) night (11pm to 5am) KSI crashes; (e) passenger crashes; (f) passenger KSI crashes; (g) alcohol (BAC>0.00) crashes; (h) alcohol (BAC>0.00) KSI crashes.

The trends in different sub-types of novice driver crashes and KSI crashes over time are depicted in Fig 3 and summary statistics are presented in Table 2. For single-vehicle crashes involving novices per 10,000 novice licences [Fig 3(a)], a change in pre and post trends was found, showing a decline at a rate of 1.0% per month or 11.5% per year following the new GLS. This finding was also observed for single-vehicle KSI crashes [Fig 3(b)], with these decreasing at a rate of 0.4% per month or 4.5% per year.

**Table 2:** Summary statistics of types of crashes^a^ and KSI crashes^b^ involving novice drivers per 10,000 novice licences in Queensland, Australia.

Time Series	Trend Pre New GLS				New GLS shift				Change in Trend Post New GLS			
	Estimate	95% CI		P	Estimate	95% CI		P	Estimate	95% CI		P
(a) Single-vehicle crashes	0.0010	0.0000	0.0022	0.0575	-0.0509	-0.1493	0.0475	0.3120	-0.0107	-0.0132	-0.0082	<.0001
(b) Single-vehicle KSI crashes	0.0009	-0.0005	0.0024	0.2050	-0.1264	-0.2475	-0.0053	0.0426	-0.0047	-0.0072	-0.0023	0.0002
(c) Night crashes	0.0010	-0.0005	0.0025	0.1834	-0.0316	-0.1653	0.1021	0.6442	-0.0144	-0.0178	-0.0110	<.0001
(d) Night KSI crashes	0.0024	0.0003	0.0045	0.0232	-0.1694	-0.3407	0.0019	0.0544	-0.0100	-0.0135	-0.0065	<.0001
(e) Passenger crashes	-0.0018	-0.0028	-0.0008	0.0006	-0.0685	-0.1571	0.0201	0.1322	-0.0095	-0.0118	-0.0073	<.0001
(f) Passenger KSI crashes	-0.0006	-0.0020	0.0008	0.3854	-0.1609	-0.2781	-0.0437	0.0079	-0.0038	-0.0062	-0.0014	0.0020
(g) Alcohol crashes	0.0035	0.0015	0.0055	0.0008	0.1335	-0.0451	0.3121	0.1445	-0.0191	-0.0236	-0.0146	<.0001
(h) Alcohol KSI crashes	0.0041	0.0014	0.0068	0.0030	-0.0275	-0.2507	0.1957	0.8096	-0.0120	-0.0166	-0.0074	<.0001

^a^ 1 July 1999 to 30 June 2012;

^b^ 1 July 1999 to 31 December 2013

Differences in pre and post trends in novice night crashes [Fig 3(c)] and night KSI crashes [Fig 3(d)] were also found, indicating declines following the new GLS at a rate of 1.3% per month or 15.9% per year and 0.8% per month or 9.1% per year, respectively. Likewise, changes in pre and post trends in novice passenger crashes [Fig 3(e)] and passenger KSI crashes [Fig 3(f)] were found, with both showing declines following the new GLS: 1.1% per month or 13.5% per year and 0.4% per month or 5.3% per year, respectively.

Changes in pre and post trends in novice alcohol crashes [Fig 3(g)] and alcohol KSI crashes [Fig 3(h)] also were found, indicating declines following the new GLS at the rate of 1.5% per month or 18.6% per year and 0.8% per month or 9.4% per year, respectively.

#### Trends in crash casualties

The analyses of overall trends in novice crashes, KSI crashes and their sub-types were repeated focusing on the number of casualties involved. Summary statistics are included in Table 3. The same patterns of differences in pre and post trends were found to those above (and therefore the figures are not included).

**Table 3:** Summary statistics of trends in casualties in crashes^a^, KSI crashes^b^ and subtypes of crashes^a^ and KSI crashes^b^ involving novice drivers in Queensland, Australia.

Time Series	Trend Pre New GLS				New GLS shift				Change in Trend Post New GLS			
	Estimate	95% CI		P	Estimate	95% CI		P	Estimate	95% CI		P
Novice driver crash casualties^c^	0.0035	-0.0005	0.0011	0.5252	0.1335	-0.0473	0.0953	0.5093	-0.0191	-0.0137	-0.0101	<.0001
Novice driver KSI crash casualties^c^	0.0013	0.0002	0.0024	0.0170	-0.1241	-0.2131	-0.0351	0.0070	-0.0057	-0.0075	-0.0039	<.0001
Proportion novice driver crash casualties	-0.0007	-0.0012	-0.0002	0.0061	0.0172	-0.0267	0.0611	0.4443	-0.0017	-0.0029	-0.0006	0.0025
Proportion novice driver KSI crash casualties	-0.0005	-0.0012	0.0002	0.1891	-0.0359	-0.0974	0.0256	0.2547	-0.0001	-0.0014	0.0012	0.8680
Novice driver single-vehicle crash casualties^c^	0.0005	-0.0008	0.0017	0.4718	0.0039	-0.1054	0.1133	0.9438	-0.0106	-0.0134	-0.0078	<.0001
Novice driver single-vehicle KSI crash casualties^c^	0.0005	-0.0011	0.0020	0.5537	-0.1454	-0.2720	-0.0188	0.0255	-0.0036	-0.0062	-0.0010	0.0067
Novice driver night crash casualties^c^	0.0008	-0.0010	0.0025	0.4035	0.0312	-0.1262	0.1886	0.6980	-0.0151	-0.0191	-0.0111	<.0001
Novice driver night KSI crash casualties^c^	0.0019	-0.0005	0.0043	0.1290	-0.1757	-0.3766	0.0252	0.0884	-0.0090	-0.0131	-0.0049	<.0001
Novice driver passenger crash casualties^c^	-0.0015	-0.0026	-0.0005	0.0053	0.0038	-0.0895	0.0971	0.9372	-0.0104	-0.0128	-0.0080	<.0001
Novice driver passenger KSI crash casualties^c^	-0.0011	-0.0026	-0.0005	0.1823	-0.1208	-0.0895	0.0971	0.0678	-0.0034	-0.0128	-0.0080	0.0121
Novice driver alcohol crash casualties^c^	0.0028	0.0005	0.0051	0.0168	0.1924	-0.0134	0.3982	0.0688	-0.0200	-0.0252	-0.0148	<.0001
Novice driver alcohol KSI crash casualties^c^	0.0029	-0.0001	0.0060	0.0623	-0.0002	-0.2520	0.2517	0.9989	-0.0110	-0.0161	-0.0059	<.0001

^a^ 1 July 1999 to 30 June 2012;

^b^ 1 July 1999 to 31 December 2013;

^c^ per 10,000 novice licences

In summary, the following trends were found in relation to casualties in crashes involving novice drivers following introduction of the new GLS:

- A decline in novice crash casualties (all) at a rate of 1.2% per month or 13.9% per year.
- A decline in novice crash casualties (all) relative to the overall licensed driver population in QLD at a rate of 0.2% per month or 2.9% per year.
- A decline in novice KSI crash casualties at a rate of 0.4% per month or 5.2% per year.
- No decline in novice KSI crash casualties relative to the overall licensed driver population in QLD.
- A decline in novice single-vehicle crash casualties at a rate of 1.0% per month or 12.1% per year.
- A decline in novice single-vehicle KSI crash casualties at a rate of 0.3% per month or 3.8% per year.
- A decline in novice night crash casualties at a rate of 1.4% per month or 17.1% per year.
- A decline in novice night KSI crash casualties at a rate of 0.7% per month or 8.5% per year.
- A decline in novice passenger crash casualties at a rate of 1.2% per month or 14.2% per year.
- A decline in novice passenger KSI crash casualties at a rate of 0.4% per month or 5.4% per year.
- A decline in novice alcohol crash casualties at a rate of 1.7% per month or 20.4% per year.
- A decline in novice alcohol KSI crash casualties at a rate of 0.8% per month or 9.7% per year.

## Discussion

Our examination of trends in crashes involving novice drivers in QLD pre and post changes to the state’s GLS identified that the new GLS was associated with declines in novice driver-related crashes and with similar rates of decline in crash casualties. The results suggested that, while crashes and casualties were declining for all drivers over the study period, the new GLS contributed to greater declines among novice drivers. While declines for novice KSI crashes and KSI crash casualties as a proportion of all such crashes in QLD did not pass the p<0.05 threshold, the declines for provisional-licensed drivers were greater than those for 25-29-year-old open licensed drivers; a more narrow age control comparison of independent drivers. Novice driver single-vehicle crashes, KSI crashes and associated casualties, a subset of crashes in which the novice drivers were most likely to be at-fault, also were found to decline.

The results of the current analyses, which controlled for exposure in terms of the licensed population (number of licences per month) supported the positive conclusions of the preliminary evaluation based on individual level exposure (person months of licensure) albeit with differing age controls and estimates of crash reductions [6]. Of comparable results across the two evaluations, the preliminary evaluation estimated a 13% reduction in novice driver KSI crashes when including 25–35 year-old drivers as the age control. A 15% or less reduction in provisional-licensed driver KSI crashes also was predicted. The current findings in fact showed a 13% decline per year in all injury crashes involving novices (i.e. medical treatment to fatal), whereas the decline in KSI crashes was more modest at 5% per year. Proportional to the licensed driver population in QLD, these findings reduced to 3% for all injury crashes and no difference in KSI crashes. More narrowly focusing on provisional-licensed drivers, the decline in all injury crashes per year was 9% and for KSI crashes near 4%, which were approximately 3% and 1% greater than the respective declines for 25–29 year-old open-licensed drivers.

By further examining the novice driver trends by crash types, the results suggested that the night passenger restriction was a key contributor to the reductions, given that night and passenger crashes were found to reduce by almost 16% and 14% respectively each year, and respective KSI crashes by 9% and 5% per year; with equivalent findings for respective reductions in casualties. Moreover, declines of around 20% in novice alcohol-involved crashes and respective casualties and declines of around 10% in novice alcohol-involved KSI crashes and respective casualties were found immediately following introduction of the new GLS, despite no change in the zero alcohol restriction at that time. This suggests there might have been an associated impact of the night passenger restriction on novice alcohol crashes. Recent self-report research in the United States is consistent with this finding: graduated driver licensing systems that were more restrictive generally (i.e. not just in relation to alcohol) were found to be associated with reduced risky alcohol-use behaviours as well as alcohol-related risky driving and passenger behaviours [10].

It is also noteworthy that previous self-report research in Queensland indicated that the 100-hour supervised driving requirement might have resulted in delayed provisional licensure, such that older age (greater maturity) at this time might be implicated in the improvements found [11]. There have also been reports of cultural shifts in young people delaying or potentially forgoing licensure elsewhere in Australia and internationally [12–14]. However, additional analyses (reported elsewhere [15]), found no significant differences in the mean, median or upper and lower quartile age between the provisional-licensed drivers pre and post the changes to QLD’s GLS. Therefore, no evidence of delayed licensure or a maturity effect was determined in the current study; albeit this does not account for any potential forgoing of licensure.

Despite the positive findings identified, as noted in the introduction, provisional licence holders nonetheless remain substantially over-represented in QLD crash statistics, suggesting further strengthening of the GLS is warranted. Research on fatal crashes in the United States has demonstrated that substantial gains can be made with stronger graduated driver licensing systems, with 30–55% greater reductions for strong versus weaker systems [16–17]. Based on the standards applied in that US research, QLD’s GLS would be rated as “fair” only [17]. The night passenger restriction in QLD is limited compared to passenger restrictions that apply at all times of day and restrictions from any unsupervised night driving, with these identified as among the most effective GLS components [18–20]. Night restrictions in particular have been associated with a 16–59% reduction in night crashes and a 35–36% reduction in night KSI crashes, as well as a 5–25% reduction in day-time crashes [4]. Recent research examining fatal crashes has also identified the need to ensure that the night restrictions capture the peak crash injury time periods to have maximum impact, particularly the early to late evening hours [19], not currently included in QLD’s GLS.

While potential contributions of other features of QLD’s new GLS are difficult to isolate, a recent review found international evaluations provide strong support for a minimum learner period of 12 months and developing support for the relatively high number of 100 supervised driving hours required in QLD [4]. However, the review found limited evidence for restrictions on phone use and a lack of support for high-powered vehicle restrictions. The review also identified some support for raising the minimum provisional driving age to 18 years, however cautioned a need for exemptions and/or additional supports for those who might otherwise be geographically and economically disadvantaged; with lack of access to licensing potentially compounding further disadvantage [21–22]. Modelling research could be undertaken to confirm the potential for more substantial road crash and trauma reductions in QLD should reforms be made to align with international best practice.

### Strengths and limitations

This is the first full evaluation of an Australian graduated driver licensing system, which commences from age 17, applies through to at least age 25 and includes a much longer provisional period compared to international systems: confirming that graduated licensing can be effective in late licensing age jurisdictions. The evaluation also confirmed that the declines in crashes and KSI crashes over time were associated with corresponding declines in crash casualties (including the novice drivers as well as other road users involved in their crashes). Analysis strengths of the current evaluation include the use of population level data, the interrupted time series approach, and control for exposure to crashes in terms of the number of licensed drivers by each licence type on a month-by-month basis. While this approach adjusted for potential differences in licensing rates over time, including potential declines in response to strengthening GLS policies [23], any differences in the amount of driving undertaken by individuals over time could not be captured due to the lack of reliable driving exposure data.

A limitation in interpreting these findings relates to the timing of the introduction of the new GLS at a time now considered to mark a global economic downturn [8]. Research on the link between economic downturns and reduced road crashes, including some modelling with data on young males, suggests that youth might be particularly affected due to assumed greater difficulty in gaining employment and thereby reduced driving exposure [8]. This concern was partly addressed by limiting some comparisons to only the youngest cohorts of recent licencees. It was found that provisional licensed drivers (who are predominantly aged under 25 years) experienced greater declines in all crash outcomes following the new GLS compared to 25–29 year-old open licensed drivers. This was despite the potential for those licensed under the new GLS to have some carryover crash-reduction benefits when first graduating to an open licence. Future research could seek to model potential links with relevant economic datasets (such as employment data and sales of alcohol, fuel, old and new vehicles, for example) for more specific examination of any differential impacts.

A further limitation that could not be directly addressed was the potential impact of a short-term divergence in the reporting of injury severities during the evaluation period. In 2006, a new incident recording system was implemented by Queensland Police. During the transition period the injury severity for some casualties was not recorded by police in the usual way and therefore sourced through other means such as medical records. This administrative change may have contributed to a drop in serious injury crashes in 2006–2007, following increases in prior years. As including the 2006–2007 serious injury data was a more conservative approach, we chose to retain these data.

## Conclusions

In conclusion, we found that the new GLS in QLD was associated with declines in overall novice driver crashes and novice driver fatal and serious injury crashes, with similar rates of declines confirmed for associated crash casualties. While identified declines when analysing all crashes were often substantial, comparative results for fatal and serious injury crashes were moderate and did not meet p<0.05 criterion when examined proportional to the QLD licensed driver population. Results suggest the night passenger restriction was a key contributor to these declines, including declines in both night and passenger crashes, and potentially associated declines in alcohol crashes. However, young novice drivers continue to be substantially over-represented in QLD crash statistics. Further strengthening of the GLS in keeping with international best practice has potential to result in the prevention of considerable more deaths and debilitating injuries. Further research directions include exploring potential confounding influences of the economic downturn for specific age and gender groups and modelling the potential for additional casualty reductions by further strengthening QLD’s GLS.

## Supporting information

S1 Dataset (CSV)
